# Supplementary material for: Aged G Protein-Coupled Receptor Kinase 3 (Grk3)-Deficient Mice Exhibit Enhanced Osteoclastogenesis and Develop Bone Lesions Analogous to Human Paget’s Disease of Bone
Source: Cells. 2023 Mar 23;12(7):981. doi: 10.3390/cells12070981 (PMC10093054; doi:10.3390/cells12070981)

Supplemental Figure S1. Osteoclast-rich TRAP+ cortical bone lesions are seen in aged Grk3$^{-/-}$ mice and not in age-matched WT controls or in young mice – regardless of genotype. (A) Representative TRAP staining for osteoclasts (purple) of a 12 week old WT femur, (B) a 12 week old Grk3$^{-/-}$ femur, (C) a 24 month old WT femur, and (D) a 24 month old Grk3$^{-/-}$ femur. All images were obtained at 5x magnification. Black arrows show osteoclast-rich lesions in the cortical bone of Grk3$^{-/-}$ 24 month-old femur.

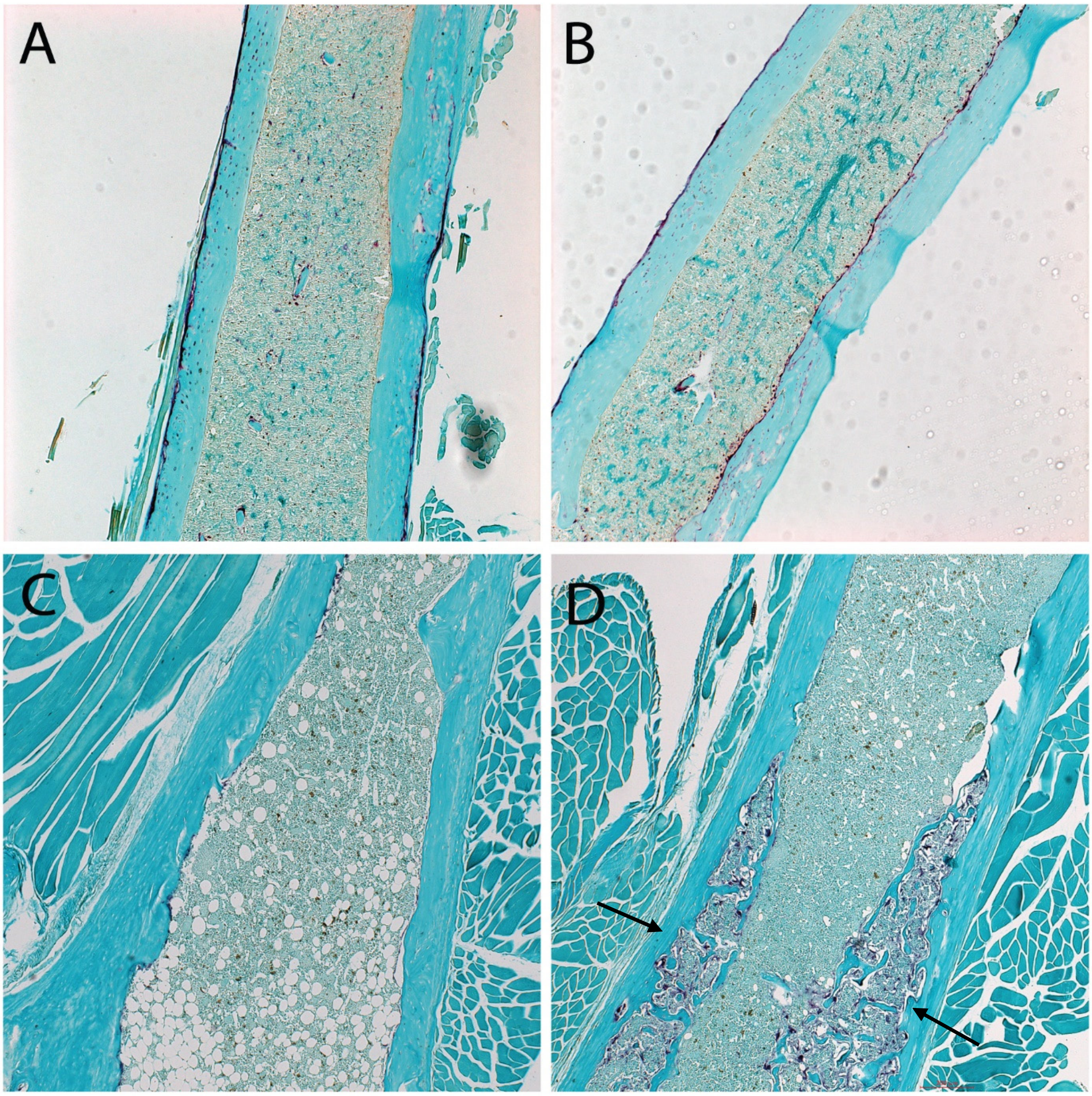

Supplement: Supplementary file 1 [file cells-12-00981-s001.zip › cells-2235773-supplementary.pdf]
